# Achieving universal reproductive health coverage for deaf women in Ghana: an explanatory study of knowledge of contraceptive methods, pregnancy and safe abortion practices

**DOI:** 10.1186/s12913-022-08323-5

**Published:** 2022-07-27

**Authors:** William Nketsia, Wisdom Kwadwo Mprah, Maxwell Peprah Opoku, Duorinaah Juventus, Michael Amponteng

**Affiliations:** 1 School of Education, Western Sydney University, Sydney, Australia; 2 Centre for Disability and Rehabilitation Studies, Department of Health Promotion and Disability Studies, Kwame Nkrumah University of Science and Technology, Kumasi, Ghana; 3 Special Education Department, United Arab Emirates University, P. O. Box 15551, Al-Ain, United Arab Emirates; 4 Ghana National Association of the Deaf, Accra, Ghana

**Keywords:** Deaf persons, Sexual and reproductive health, Policies, Culture, Religion

## Abstract

**Background:**

The first world conference on sexual and reproductive health (SRH) in 1994 helped create the awareness that reproductive health is a human right. Over the years, attempts have been made to extend services to all persons; however, lapses persist in service provision for all in need. Recently, countries have been encouraged to target minority groups in their reproductive health service provision. However, studies have rarely attempted to develop deeper insights into the experiences of deaf men and women regarding their knowledge of SRH. The purpose of this study was to develop an in-depth understanding of the knowledge of deaf persons regarding services such as knowledge of contraceptive methods, pregnancy and safe abortion practices.

**Methods:**

A sequential explanatory mixed-methods approach was adopted for this study. In the first quantitative phase, 288 deaf persons recruited from three out of the 16 regions in Ghana participated in this study. They completed a 31-item questionnaire on the main issues (knowledge of contraceptive methods, pregnancy and safe abortion practices) addressed in this study. In the second phase, a semi-structured interview guide was used to collect data from 60 participants who took part in the first phase. The key trend emerging in the first phase underpinned the interview guide used for the data collection. While the quantitative data were subjected to the computation of means, t-tests, analyses of variance, correlations and linear regressions to understand the predictors, the in-depth interviews were analysed using the thematic method of analysis.

**Results:**

The results showed a convergence between the quantitative and qualitative data. For instance, the interview material supported the initial findings that deaf women had little knowledge of contraceptive methods. The participants offered reasons explaining their inability to access services and the role of religion in their understanding of SRH.

**Conclusion:**

The study concludes by calling on policymakers to consider the needs of deaf persons in future SRH policies. The study limitations and other implications for future policymaking are discussed.

## Introduction

Sexual and reproductive health (SRH) is a key component of achieving universal health coverage, which the WHO believes is a game-changer in countries developing policies to ensure equitable access to health [1–4]. In this study, SRH refers to the state in which an individual has complete control over their physical, emotional, mental and social well-being on matters relating to their reproductive system [5, 6]. SRH is complex and constitutes four main components: sexual health, sexual rights, reproductive health and reproductive rights [6]. While sexual health refers to the ability of individuals to access information about treatment and prevention of sexually transmitted diseases, sexual rights include rights to sexual education, choose a partner, engage in consensual sex and be free from sexual abuse. Reproductive health also includes the capacity and ability of individuals to receive information about issues relating to the reproductive health system, contraceptives and safe abortion services [6]. Furthermore, reproductive rights encapsulate the decision to plan the number of children that a person wishes to have, that is, family planning, and freedom from coercion [6]. This study focuses on reproductive health and rights that prioritize issues such as knowledge of contraceptives, pregnancy-related issues and safe abortion practices as these issues are of direct cultural significance to sub-Saharan Africa [7–10].

In sub-Saharan Africa and other low- and middle-income contexts, there are cultural stereotypes around issues of SRH [11–22]. For instance, traditionally, contraceptives have been deemed unacceptable and contrary to people’s beliefs about procreating to continue or sustain lineages. This has had the consequence of people’s wealth being judged by the number of children they have. Thus, barrenness is frowned upon [17], and women who are unable to give birth are labelled *boyini* (by Twi speakers in Ghana), meaning unable to have a child. As part of marital ceremonies, prayers are offered to the ‘gods’ and ancestors to bless the couple with children. In fact, in the event of a lack of childbirth after years of marriage, women are usually blamed and suffer consequences such as being driven from their matrimonial homes [17]. In this social context, the introduction of contraceptives is perceived as alien to culture and tradition [23].

There is a corpus of literature on challenges relating to the accessibility of SRH services, especially in sub-Saharan African contexts [13, 16, 24–27]. Most studies have reported that challenges such as lack of policies [28, 29], low education levels [30], poverty [12, 24, 31, 32], culture [33, 34], stigma [8, 16] and lack of parental support [14, 35] contribute to the inability of girls and women to access SRH services. Other studies have also found that a high prevalence of teenage pregnancy is likely linked to factors such as poverty [12, 25], illiteracy [30, 36–38] and lack of awareness of contraceptive methods [15]. Furthermore, giving birth as a teenager has its own health and social drawbacks [37, 39]. Specifically, young mothers are at risk of developing poor health outcomes and struggle to cater to their children [37, 39]. Furthermore, abortion has been deemed culturally unacceptable and unlawful [33], leading to limited knowledge or accessibility of services [16].

While the factors affecting access to SRH services are broad, they are interrelated. Most of the available literature has placed a great deal of emphasis on factors affecting practices among young girls or women. However, there has only been a limited attempt at capturing both male and female voices in the SRH discourse. It is important to state here that deaf males are likely to enter into a relationship with a female who is also deaf [18–20]. This arguably supports the development of an in-depth understanding of the awareness of deaf persons on issues such as contraceptive methods, pregnancy and safe abortion services.

## Deaf persons and SRH

Deafness is a sensory disability characterized by a partial or profound inability to hear, which interferes with the reception and processing of conversational speech or loud sounds [20–22, 40]. Deaf persons most often use sign language as their preferred mode of communication [21, 22]. The WHO estimates that over five percent of the world’s population or 430 million people have some form of hearing problem or are deaf [40]. This figure is expected to increase to 2.5 billion people by 2050, with 700 million requiring hearing rehabilitation. The population of deaf persons in Ghana is estimated to be 22% of the two million persons with disabilities [41]. The high percentage of deaf persons in Ghana emphasizes the urgency for SRH needs and rights to be incorporated into national policies [40], thereby conforming with the global call in the Convention on the Rights of Persons with Disabilities for countries to mainstream the sexual and reproductive rights of persons with disabilities in healthcare policies [42]. However, globally, the unique cultural and linguistic characteristics of deaf persons has resulted in their elimination from necessary social services such as education, reproductive healthcare, recreation, transportation, employment, economic activities, etc. [18–22, 43–46]. Thus, there are ongoing international engagements and deliberations aimed at adopting innovative reforms to promote access to social services for deaf persons.

Several studies have explored the accessibility of healthcare and reproductive health services to persons with disabilities [47–54]. A qualitative study by Gartrell et al. about the reproductive health rights of Cambodian women with disabilities identified challenges encountered by these women when accessing reproductive health services, such as physical restrictions, limited access to information and communication and financial barriers [53]. A similar qualitative study by Burke et al. in Senegal discovered formidable barriers to access to health care such as finance, negative attitudes and the lack of health facilities in communities that restrict access to services [51]. Studies from Ghana have also identified limited access to information, lack of finance, negative attitudes and lack of communication as challenges that restrict access to SRH services to women with disabilities, such as those with visual impairment [47, 48] and deafness [43–45]. Deaf women are also at risk of not being able to access SRH information due to illiteracy and the inability of health professionals to communicate with them using sign language [43, 45].

Studies on SRH and deafness are inexhaustive as the current literature has generally neglected or paid very little much attention to specific conditions such as knowledge of contraceptives, pregnancy-related issues and abortion. It is useful to point out that although Ghana has adopted international policy frameworks such as the Sustainable Development Goals (SDGs), current policies on SRH such as the Adolescents’ Reproductive Health Policy and Ghana Population Policy [48] were developed with little consideration of deaf persons and those living with other impairments [43, 45]. This arguably calls for more explanatory studies to present holistic information about SRH, which could be considered in future policies. Consequently, the current study attempted to use a mixed-methods design to develop a deeper understanding of the knowledge of deaf persons regarding SRH components such as knowledge of contraceptives, pregnancy-related issues and abortion. The study was guided by the following questions:

- Which background variables could impact or predict deaf persons’ understanding of contraceptives, pregnancy-related issues and abortion?
- How knowledgeable are deaf persons when it comes to contraceptive methods, pregnancy-related issues and safe abortion practices?

## Method: Phase 1

The first phase of this study was the collection of data from a sample size of 288 participants in selected districts and areas. The sections below present information about the participants, the instrument used for the data collection, the procedures and data analysis.

### Participants

The study participants were recruited in collaboration with the Ghana National Association of the Deaf (GNAD), which is the main association promoting the well-being of deaf persons in Ghana. The research team had a meeting with the GNAD, which suggested recruitment from six districts in three regions. According to the GNAD, the regions recommended for the study covered their three main categorizations of the country: the North (Northern region), middle belt (Ahafo region) and Southern sector (Greater Accra regions). The selection would help develop a snapshot of the understanding of deaf persons across Ghana. In each region, two districts were selected for the recruitment of study participants. A simple random method was used to invite the participants. Invitations were sent to the list of members supplied to the research team by the GNAD. Every member in the study areas had an equal chance of participating in this study. Thus, those who responded favourably to the invitation were considered for recruitment.

### Instrument

A four-part questionnaire was used for the data collection. The first part collected background information (gender, age, educational qualification, marital status and religion) on the study participants. The decision to collect this information was based on a review of the literature [1, 2, 12–17, 20, 35, 38, 49, 50] and recommendations from the GNAD and experts about appropriate information to collect from deaf persons.

The second part of the questionnaire collected information relating to the participants’ knowledge of contraceptive methods. The section comprised 11 items with two dichotomous responses (yes or no). Some of the items on the scale were as follows: ‘Women can take a pill everyday’, ‘Women can have an injection every 2 or every 3 months’ and ‘A woman can take pills soon after intercourse.’ The actual mean scores (sum mean divided by the number of items) instead of the sum means were reported. All the items were positively worded. Thus, a mean score of 1 was interpreted as more knowledgeable about contraceptive, while a score of at least 1.5 was interpreted as low level of knowledge about contraceptives.

The third part of the questionnaire captured the participants’ knowledge of pregnancy-related issues. The section contained 10 items anchored on a three-point scale (1 = true, 2 = false and 3 = don’t know). Some of the items on the scale were as follows: ‘A woman can’t get pregnant because she is breastfeeding’, ‘A woman can’t get pregnant if she has sex standing up’ and ‘showering, bathing right after sex will prevent pregnancy’. Since the statements were untrue, a mean score of at most 1.5 was interpreted as a low level of knowledge, 1.6 to 2.4 as more knowledgeable and at least 2.5 as ambivalent towards pregnancy.

The fourth section comprised statements relating to the participants’ knowledge of safe abortion services. The section included 10 items, which were also anchored on a three-point scale ranging from 1 = agree, 2 = disagree and 3 = don’t know. Some of the items on the scale were as follows: ‘Many young deaf girls and women in Ghana engage in abortion’, ‘A woman can request an abortion from a qualified doctor if she needs it’ and ‘All forms of abortion are murder’. Since the statements were untrue, a mean score of at most 1.5 was favourable towards abortion, 1.6 to 2.4 was unfavourable towards abortion and at least 2.5 was ambivalent towards abortion services.

The questionnaire was reviewed by three academics and three members of the deaf community in Ghana and the USA. They made useful suggestions, which were incorporated in the draft used for the data collection. For example, they suggested reducing and ordering the anchors, removing some demographics (such as employment status) and rewording some of the questions.

The questionnaire was then piloted among 10 participants from different regions. The pilot yielded the following reliability scores: knowledge of contraceptive methods (0.87), knowledge of pregnancy-related issues (0.70) and knowledge of abortion (0.73). In this study, the reliability scores, as computed using Cronbach’s alpha, were as follows: knowledge of contraceptive methods (0.77), knowledge of pregnancy-related issues (0.72) and knowledge of abortion (0.72).

### Procedure

This study and its protocols were first approved by the Human Research and Ethics Review Committee at the School of Medical Sciences, Kwame Nkrumah University of Science and Technology (CHRPE/AP/375/16). The protocols were subsequently approved by the GNAD and Ghana Health Services before being used for this study. Following approval, the research team communicated to the GNAD their interest in heterogeneous participants to take part in the study. Consequently, the national office of the GNAD suggested to the research team the areas where data could be collected. After a consensus was reached on the study areas, a formal letter was sent by the GNAD to their regional and district representatives to invite their members to be part of this study.

The GNAD provided the research team with the full list of potential participants. The team then sent formal emails, texts and WhatsApp video statements to explain the study and invited the prospective participants. WhatsApp video was used to provide a signed explanation of the study to potential participants who might have found it difficult to read. The formal statements clearly explained to the participants that they had the right to withdraw from the study at any time and without consequences. In addition, assurance was provided to them that their identity would remain strictly confidential throughout the research process. All the participants provided written consent before taking part in the study.

The research team recruited and trained two graduate students who were proficient in English and Ghana Sign Language to collect data from the study participants. The research team reached an agreement with the participants to visit the GNAD to complete the questionnaire at a time convenient to them. They delivered printed copies of the questionnaires, which were then completed by the participants. Those who were unable to read were provided with support by the research team to complete the questionnaires. The data were collected on weekends at the GNAD district office and took place from June 2016 to December 2018. The participants were reimbursed for costs relating to transportation to and from the GNAD district office as well as breakfast and lunch.

### Data analysis

The completed questionnaires were entered into Excel for cleaning before being transferred to SPSS for data analysis. Due to the number of study participants, we assumed that the data were normally distributed, thus requiring parametric tests [55]. Research question one pertaining to the quantitative data was answered as follows: the computation of mean scores, t-tests and analysis of variances (ANOVA) and linear regression to understand the predictors of SRH.

The mean scores were computed to understand the participants’ level of knowledge of SRH. Afterward, t-tests (for demographics with two levels) and ANOVA (for demographics with at least three levels) were computed to understand the differences between the participants on the three measures. For the t-test, the assumption of homogeneity of variance was checked using Levene’s test, which showed no violation for any of the computations [56, 57]. The weight of the results was assessed using Cohen’s *d*, which was interpreted as follows: small (0.10 to 0.29), moderate (0.30 to 0.49) and large (0.50 to 1.0). For the ANOVA, in the event of a violation of homogeneity of variance, the results of the Welch statistics were reported [56]. Here, the effect size was assessed using partial eta squared, which was interpreted similarly to Cohen’s *d*.

Third, the predictors of SRH were computed using linear regression. However, prior to this, the relationship between the measures was assessed using Pearson’s moment correlation, yielding the following results: (0.10 to 0.29), moderate (0.30 to 0.49) and large (0.50 to 1.0) [57]. Thereafter, three model hierarchical regressions were computed to determine the predictors for each of the measures. The following assumptions were assessed to ensure they were not violated: linearity, multicollinearity and homoscedasticity [56, 57].

## Method: Phase II

The study took the form of a sequential explanatory mixed-methods design. Under this approach, data were collected in phases to offer an in-depth explanation of the given phenomena [58–60]. Thus, after the first phase, follow-up interviews were conducted with some of the study participants, giving them the opportunity to clarify and provide an in-depth explanation of key trends emerging in the first phase [58–60].

The participants of this second phase were drawn from initial phase. A meeting was held between the research team and the GNAD about the recruitment strategy and procedures. There was consensus that at least 10 participants would be recruited from each district for this phase. Random invitations were sent to the participants, and once the required number was reached, the interviews were scheduled. It was agreed that there would be one focus group discussion made up of seven people and that the remaining three would be engaged in one-on-one interviews.

An interview guide was developed based on key issues emanating from the first phase [61, 62]. The issues covered were the participants’ understanding of contraceptive methods, pregnancy and safe abortion practices. Some of the questions included *‘In the first phase, the results showed that most deaf persons had little knowledge about contraceptive methods. What do you think about this?* Under each of the components, the participants were asked to comment on a question, such as *‘The age of participants was found to influence knowledge about pregnancy. We noted that the older persons were more knowledgeable about pregnancy than the young participants. What is your view on this?’.*

The video recordings were transcribed in text format by two research assistants. The transcribed data were then shared with some of the participants to check whether their views were captured correctly for possible iteration [63]. In addition, WhatsApp video calls were made to other participants to discuss the key themes emerging from the interview data. The participants indicated that they were satisfied with the information and that it could be used in the reporting.

The next stage was the data analysis, where we conducted a thematic analysis based on recommendations by Braun and Clarke [61, 62]. The steps of the data analysis were reading, coding, mapping and charting, sorting and writing the draft [61, 62]. The key issues from the first phase guided the development of the interview guide: knowledge of contraceptive methods, knowledge of pregnancy-related issues and knowledge of abortion-related issues. First, the transcribed data were read several times by authors two, three and four to gain familiarity with the content. According to Creswell and Miller, accountability and transparency in qualitative analysis are very important in enhancing the rigor of the findings [63]. During the analysis, the authors coded the data from one interview before meeting to discuss the assigned codes. Here, similarities and differences in the codes were identified, and a consensus was reached among the authors. Author four continued to code all the transcribed data. Second, the codes were sorted and mapped under sub-themes. This enabled the authors to map common ideas and identify areas of disagreement among the participants. The ideas regarding key issues or topics were grouped under themes [64]. Finally, the quotes explaining the categories were extracted and transferred onto a new file. Author three continued to write the results section, which was shared with all the authors for reading and approval.

## Results: Phase I

A total of 360 questionnaires were distributed to prospective participants, and 288 were returned, representing an 80% return rate. In terms of gender, 75% were female, while 25% were male. Regarding age, 59% of the participants were between 17 and 25 years compared to 15% who were at least 36 years. With respect to education, 50% of the participants had basic qualifications, while 11% indicated that they had tertiary qualifications. Furthermore, 71% indicated that they were married, while 29% were single. Regarding religion, 68% stated that they were Christian, while 32% specified that they were Muslim (see Table 1).

**Table 1:** Association between demographics and knowledge of reproductive health

*N* = 288	Sample (%)	Contraceptives	Pregnancies	Abortion
**Gender (*n* = 284)**				
Male	72 (25%)	1.80 (.58)	2.23 (.58)	2.14 (.62)
Female	212 (75%)	1.91 (.41)	2.24 (.56)	2.24 (.56)
*t*		.82#	-.07	-1.27
Cohen’s *d*		.13	.01	.18
**Age (*n* = 284)**				
17–25 years	167 (59%)	1.73 (.42)^a^	2.18 (.56)^a^	2.17 (.52)^a^
26–35 years	74 (26%)	1.74 (.53)^a^	2.20 (.53)^b^	2.20 (.63)^a,b^
36 years and above	43 (15%)	1.87 (.51)^a^	2.50 (.56)^c^	2.42 (.61)^b,c^
*F*		1.52	5.55##**	3.09##*
Partial eta squared		.01	.04	.02
**Educational status (*n* = 284)**				
Basic	141 (50%))	1.71 (.37)	2.17 (.55)	2.16 (.56)
Secondary	112 (39%)	1.79 (.56)	2.28 (.55)	2.25 (.54)
Tertiary qualification	31 (11%)	1.82 (.42)	2.36 (.59)	2.35 (.70)
*F*		1.35	2.22	1.83
Partial eta squared		.01	.02	.01
**Marital Status (*n* = 284)**				
Single	82 (29%)	1.84 (.55)	2.36 (.58)	2.30 (.61)
Married	202 (71%)	1.72 (.42)	2.19 (.55)	2.18 (.55)
*t*		1.88#*	2.35**	1.63*
Cohen’s *d*		.28	.31	.21
**Religion (*n* = 278)**				
Christianity	188 (68%)	1.77 (.50)	2.16 (.58)	2.17 (.51)
Muslim	90 (32%)	1.72 (.37)	2.38 (.46)	2.29 (.65)
*t*		.89	-3.32#**	-1.54#*
Cohen’s *d*		.11	.39	.22

^*^p < .05; **p < *.01;*

superscripts^(abc)^ = significant difference between participants;

^#^ = violation of assumption of homogeneity for t-test;

^##^violation of assumption of homogeneity for ANOVA and reporting of Welch statistics

### Association between background variables and reproductive health

The results showed the following means scores: knowledge of contraceptive method (*M* = 1.76; *SD* = 0.46), knowledge of pregnancy-related issues (*M* = 2.23; *SD* = 0.56) and knowledge of safe abortion practices (*M* = 2.22; *SD* = 0.57). These results suggest that the participants were less knowledgeable about contraceptives, more knowledgeable about pregnancy-related issues and less inclined towards abortion.

The associations between the participants’ profile and knowledge of reproductive health matters were computed using a t-test and analysis of variance (ANOVA) (see Table 1). While the t-tests were computed for demographics with two levels (e.g. gender), the ANOVAs were calculated for demographics with at least three levels (e.g. age).

In relation to the t-tests, the results showed a relationship between the participants in terms of religion and marital status only. Regarding religion, differences were reported between the participants in terms of their knowledge of pregnancy- (*t* (275) = -3.32, *p* = 0.001) and abortion-related issues (t (275) = -1.54, p = 0.05). Specifically, the participants who indicated that they were Muslim were more knowledgeable on pregnancy-related issues and had less favourable attitudes towards abortion than those who self-described as Christians. However, the effect sizes were very small: knowledge of pregnancy (Cohen’s* d* = 0.39) and abortion-related issues (Cohen’s *d* = 0.22).

In relation to marital status, a significant relationship was found between the participants on all the three measures: knowledge of contraceptive methods (*t* (282) = 1.88, *p* = 0.03, Cohen’s *d* = 0.28), knowledge of pregnancy-related issues (*t* (281) = 235, *p* 0.01, Cohen’s *d* = 0.31) and knowledge of abortion (*t* (281) = 1.63, *p* = 0.05, Cohen’s *d* = 0.21). To elaborate, those who indicated that they were single were less knowledgeable about contraceptive methods, more knowledgeable about pregnancy-related issues and had less favourable attitudes towards abortion than those who were married. Once again, the effect sizes were very small.

The results of the ANOVAs showed a significant difference between the participants on age with respect to two measures. In relation to knowledge of pregnancy-related issues, those who indicated that they were at least 36 years were more knowledgeable than others *F* (2, 280) = 5.74, *p* = 0.004, partial eta squared = 0.04. Specifically, a post-hoc comparison using the Tukey HSD showed that participants who were at least 36 years old differed from those between the ages of 17 and 25 and 26 and 35 years.

On knowledge about abortion, participants who were at least 36 years old had less favourable attitudes towards abortion than those who were younger, *F* (2, 280) = 3.48, *p* = 0.03, partial eta squared = 0.02. A post-hoc comparison using the Tukey HSD showed that participants who were at least 36 years differed from those who were between 17 and 25 years.

### Predictors of knowledge of reproductive health

Pearson’s moment correlation was computed to understand the relationship among knowledge of contraceptives, pregnancy and abortion-related issues. The computation showed a positive correlation between the variables: contraceptive and pregnancy (*r* = 0.46, *p* = 0.001), contraceptive and abortion (*r* = 0.41, *p* = 0.001) and abortion and pregnancy (*r* = 0.60, *p* = 0.001).

A three-way linear regression model was computed to understand the predictors of the measures (see Tables 2, 3 and 4). In the first model, the demographic variables were regressed on knowledge of contraceptive methods, and the results emerged as insignificant, F (5, 272) = 1.46, *p* = 0.20. The demographic variables made a non-significant contribution of three percent to the variance in the knowledge of contraceptives (see Table 2).

**Table 2:** Demographics regressed on knowledge of contraceptives

Category	*B*	*S. E*	*Beta*	*t*	p*,**
Gender	-.66	.70	-.06	-.95	.34
Age	.15	.50	.02	.30	.77
Educational status	.41	.49	.06	.85	.40
Marital status	-1.06	.79	-.10	-1.35	.18
Religion	-.58	.65	-.05	-.89	.37

^*^p < .05; **p < .01

In the second model, the demographic variables made a significant contribution of seven percent to the variance in knowledge of pregnancy-related issues, *F* (5, 271) = 4.00, *p* = 0.002 (see Table 3 for details). The best and most significant predictor of knowledge of pregnancy was religion (beta = 0.17, *p* = 0.006). The more religious the participants were, the higher their level of knowledge about pregnancy.

**Table 3:** Demographics regressed on knowledge of pregnancy-related issues

Category	*B*	*S. E*	*Beta*	*t*	p*,**
Gender	.001	.75	.001	.002	.10
Age	.98	.53	.13	1.84	.07
Educational status	.14	.52	.02	.28	.78
Marital status	-.97	.86	-.08	-1.14	.26
Religion	1.97	.71	.17	2.78	.006**

^*^p < .05; **p < .01

With respect to the third model, the demographic variables made a significant contribution of four percent to the variance in abortion, *F* (5, 271) = 0.2.37, *p* = 0.04 (see Table 4). Individually, only age was noted to have made a significant contribution to the variance in abortion (beta = 0.14, *p* = 0.05). This arguably suggests that resistance towards abortion increased with age.

**Table 4:** Demographics regressed on knowledge of abortion

Category	*B*	*S. E*	*Beta*	*t*	p*,**
Gender	.85	.78	.07	1.09	.28
Age	1.06	.55	.14	1.94	.05*
Educational status	.21	.54	.03	.39	.69
Marital status	-.47	.88	-.04	-.54	.59
Religion	.97	.72	.08	1.35	.18

^*^p < .05; **p < .01

## Results: Phase II

In all, 60 participants took part in this study; six focus group discussions (*n* = 42) and 18 face-to-face interviews were conducted. Eighteen participants were male compared to 42 who were female (see Table 5). All the participants signed an informed consent form before taking part in the study.

**Table 5:** Demographic characteristics of study participants

Categories	Sample (*N* = 60)
**Mode of participation**	
Focus group	42
One-on-one interview	18
**Gender**	
Male	18
Female	42
Age (years)	
17–25	33
26–35	10
36–45	11
46 +	6
**Religion**	
Christian	34
Muslim	22
Other	4
**Educational level**	
Primary level	31
High school level	16
Tertiary qualification	13
**Employment**	
None	26
Student	5
Self-employed	19
Public service	10
**Marital Status**	
Single	26
Married	29
Divorced	5

Some participants were invited to comment on a few of the trends emerging from the first phase, such as their perception of contraceptive methods, pregnancy and knowledge of abortion. They also commented on the key differences noted under each of them.

### Knowledge of contraceptive methods

Almost all the participants agreed that deaf persons had little knowledge about contraceptive methods. Most of them believed that this was because of their inability to access information on SRH. In particular, healthcare workers were blamed by some participants for their lack of understanding of deaf people and their mode of communication. A participant remarked as follows:

In trying to explain the kind of information on SRH issues, I want healthcare workers to be patient with me. But I am often misunderstood by the hearing people who are experts in giving information and services on SRH issues because they do not understand sign language, which is our mode of communication. (Female, Focus Group Participant 5, District A)

Low educational attainment among deaf people was another major barrier to access to SRH services, as highlighted in the quote below.

Some of us never went to school before and, as such, could not sign and write well. Therefore, it becomes a problem when we are inquiring about information and services on any of the SRH services from health workers because of a lack of sign language interpreters (Male, Focus Group Participant 3, District C).

Inadequate access to SRH services seemed to have exposed deaf people to many SRH problems. According to the participants, many deaf women and girls engaged in unprotected sex, which often led to unwanted and mistimed pregnancies and sexually transmitted diseases. They lacked knowledge about family planning methods and good personal hygiene because of a lack of awareness of these issues. Because of a lack of knowledge on SRH issues among deaf people, ‘most of the deaf, especially women and girls, used inappropriate methods to deal with unwanted pregnancies and abortion’ (Male, Interview Participant 2, District F). Others said as follows:

Deaf people do not understand or know the consequences of unprotected sex and other risky behaviour. I heard that some deaf girls wanted to have an abortion when they had unwanted pregnancies, but they could not do it because of fear of their parents and negative comments that would come from their peers, and as such, they stopped schooling. Some of them use the wrong methods because they do not know [lack of knowledge and fear of parents]. (Female, Focus Group Participant 5, District D)

Family planning is also a problem within the deaf community because many deaf people lack knowledge about contraceptives, so most deaf people do not know how to prevent pregnancy, and this causes a of lot problems relating to unwanted pregnancy among deaf women and girls. I hear some are suffering from diseases. (Female, Focus Group Participant 5, District A)

Almost all the participants agreed that married persons were more knowledgeable than single individuals when it came to awareness of contraceptive methods. They attributed this difference to the experience of married persons, who were likely to have experienced pregnancy or childbirth and may have encountered professionals who would have advised them about contraceptives and safe sex. Others also said that the partners of deaf persons could educate them or engage them in conversations about contraceptive methods. The participants asserted that some men were very supportive and contributed positively towards improving deaf women’s knowledge of contraceptive. The men, according to these participants, allowed their female partners to use protection and seek safe abortion services. A male participant who supported this view indicated that some of the men educated deaf women on how to avoid unwanted pregnancy and unsafe abortion. ‘Some men do talk about what they know and hear to deaf women. For example, some men participated in workshops on reproductive measures and, when they returned, shared [what they learnt] to their partners’ (Male, Interview Participant 2, District C). Another participant shared as follows:

Yes, married deaf persons are safe! A majority of deaf people, especially single girls and boys, engage in unprotected sex because they feel it is more pleasurable than using condoms, and they like it without using protection. They don’t use any protection. This expose them to sexually transmitted diseases, but because they are unaware of the effects, they don’t care. (Male, Interview Participant 2, District A)

### Knowledge of pregnancy-related issues

There was mixed reaction from the participants in terms of their knowledge of pregnancy. While some participants agreed with the findings of the first phase, most disagreed that deaf persons were more knowledgeable about pregnancy-related issues. Those who supported the finding that deaf women were more knowledgeable about pregnancy attributed their reasoning to regular education received from churches and NGOs on unwanted pregnancy. Some of the organizations identified by the participants were health centres (such as hospitals and clinics), churches, schools, the GNAD, Planned Parenthood Association of Ghana and the Peace Corps. According to the participants, a wide range of services were offered by these organizations, including pregnancy prevention, especially among school children, the prevention of STIs such as HIV and gonorrhoea and personal hygiene management. Some of the participants either visited these organizations for SRH services or attended their educational programmes both in deaf schools and their communities. Two focus group participants, who benefitted from health workers and the Peace Corps, respectively, stated as follows:

Yes, I attended a programme by the nurses who visited the community. They did practical demonstrations with dummies. They also used pictures with storylines one after the other, and that helped me partially understand whatever education they were giving for me to control my birth spacing. (Female, Focus Group Participant 6, District F)

At school, the Peace Corps educated pupils on condoms, menstrual cycle, pregnancy prevention, HIV and AIDS and reproductive systems, and they also trained them on how to use condoms and taught us about the organs of the male reproductive system and the female reproductive system. (Female, Focus Group Participant 5, District B)

Some of the participants also mentioned that they had learned about pregnancy-related issues from books, dramas and social media. They said that pregnancy was a matter of life and death, which pushed them to read more about it. A focus group participant stated that ‘Some deaf women cannot get information on reproductive health from workers, and instead, they only do research and read about reproductive health issues on the Internet and WhatsApp’ (Female, Focus Group Participant 6, District D). A male interview participant said that ‘I learned a lot about reproductive health from drama and reading books. They are good sources, but not all are accessible’ (Male, Focus Group Participant 1, District B).

Conversely, those who disagreed pointed to various reasons affecting the ability of deaf women to accesses pregnancy-related information. According to the participants, deaf people experienced many challenges when accessing SRH services from providers that do not use accessible formats to communicate with deaf people. A focus group participant, for example, said that ‘Ghana Health Service did not provide interpreters on its programmes on TV3, GTV, Africa TV, etc., so it makes it difficult for the deaf to get information on SRH issues from them’ (Female, Focus Group Participant 7, District D).

Overall, all the participants agreed that religion and marital status played a major role in improving pregnancy-related knowledge. For instance, most participants indicated that religious bodies did their best to provide accessible information to deaf persons generally. According to some of the participants, churches played a vital role in providing information on SRH issues for many deaf people. A participant who benefited from this resource indicated that churches sometimes liaised with health workers to organize SRH programmes to educate their members during camp meetings. ‘They [churches] offer some information and services on some of the issues related to SRH during church camps when they invited health workers’ (Female, Focus Group Participant 4, District B). However, responses from some of the participants suggested that although churches are vital sources of information on SRH issues, the information provided is unlikely to be adequate and beneficial to deaf people. An adult male interview participant stated that ‘If there could be any information on SRH issues, it only happens during church camps, but it is very rare and inadequate’ (Male, Interview Participant 1, District E). Similarly, an adult male interviewee indicated that ‘They [churches] do not provide information on the sensitive issues that I would like to know more about, such as the issue of abortion’ (Male, Interview Participant 2, District F).

### Knowledge of abortion-related issues

There was a consensus among them that abortion was culturally, religiously and legally unacceptable in the Ghanaian context, and they were not surprised by the results of the first phase. As such, it would be very difficult for deaf women, in particular, to request such a service. For example, most participants would not recommend it due to religious reasons and the likelihood that people would engage in unprotected sex. One of the participants explained that ‘abortion should not be allowed under any condition. It is a bad thing that can harm the woman, and also, it is a sin to take someone’s life’ (Male, Interview Participant 2, District A). It ‘is a wicked act of killing babies; therefore, it is not a valuable option for women’ (Female, Focus Group Participant 5, District A).

However, the participants championed the existence of safe abortion practices. Many of them indicated that the existence of safe abortion services, however, was expensive and inaccessible to deaf women. This is partly because abortion is not a constitutional right in Ghana, and as such, health workers charge inaccessible amounts of money for the service, making it out of reach for deaf women, who are mostly unemployed. Another reason recounted by the participants were language barriers and negative attitudes towards deaf persons, making it difficult for deaf women to access abortion services. For example, one participant said that ‘Most of us, especially women, feel shy about talking about abortion. Because of that, we are not encouraged to ask for information and services on abortion’ (Female, Interview Participant 1, District C). A male interviewee attributed the problem to communication barriers: ‘It is difficult to get information due to a lack of interpreters and communication problems, and information on abortion is inaccessible on TV’ (Female, Focus Group Participant 5, District B).

Indeed, all the participants agreed that age and religion could influence abortion acceptance. There was disagreement among the participants in terms of age groups that were more receptive of abortion. Another male interviewee reported that ‘Most deaf girls do not want to die from abortion; therefore, they do not engage in it [abortion] often, but deaf adult women engage in it more than the young girls’ (Male, Interview Participant 3, District A). However, most participants thought that young girls engaged in abortion more frequently than women because they were more sexually active than older women. A participant who supported this view reported that ‘Because the sexual edge is high [among deaf girls], abortion is more frequent among young girls than old women who have a lower appetite for sex (Female, Interview Participant 4, District A). Notwithstanding the disagreement, all the participants agreed that the religiously affiliated in Ghana were more anti-abortion. In relation to religion, there was a consensus among the participants that abortion could be harmful to the woman and that it was immoral to engage in the practice. They also expressed their unwillingness to recommend it to other deaf people. However, some agreed with safe abortion in the event the life of a family member or friend was at risk.

## Discussion

In this study, attempts were made to develop a deeper understanding of knowledge of SRH among deaf persons. Understandably, being deaf has its own drawbacks on individuals [20–22], and systemic barriers call for more affirmative policies to achieve full inclusion in society [42]. However, the findings of this study showed that deaf people may not be able to assert their SRH rights. For example, the results showed a low level of knowledge among both male and female deaf persons when it comes to contraceptive methods. This finding is consistent with that of other studies on young adolescents and their limited knowledge of contraceptive methods [37–39]. In the Ghanaian context, this is unsurprising because deaf persons are not considered in national development and planning [48]. In fact, none of the domestic policies on SRH referenced persons with disabilities, such as those who took part in this study. There is an increased potential for the participants and others with shared characteristics to engage in risky lifestyles that might expose them to sexually transmitted diseases. Policymakers could reflect on practices and engage the deaf community on matters of SRH and possible ways of educating them about contraceptives and other important issues.

The centrality of NGOs in the provision of SRH services to deaf was apparent. The results showed that some of the participants could access education or services from private organizations. Leading NGOs in service provision to the disability community operate in the areas of education and skills training [65]. This suggests that the government has been conspicuously missing when it comes to matters relating to the provision of basic services such as SRH to deaf persons. In particular, there has been limited effort by the government in the provision of basic services such as health care to deaf persons in Ghana [44, 49, 50]. However, the reliance on private sector actors for service provision has its own ramifications. In particular, they work with funding from donors; as such, the service provided has been critiqued as unsustainable [65]. In most instances, they provide interventions through projects which usually have a very short lifespan. Unfortunately, there is poor coordination of services for deaf persons amidst a lack of national guidelines on intervention programmes for the deaf community [43, 48]. Specifically, a community-based rehabilitation programme involving persons with disabilities and the community in the development of intervention programmes has been noted as an appropriate way to design formidable intervention programmes [65]. Unfortunately, Ghana is yet to develop its own community-based strategy, which could be adopted or followed by all private organizations interested in service provision to deaf persons [65]. As such, few deaf persons might receive ad hoc education on contraceptive methods, further compounding their vulnerability towards unsafe practices.

Marital status appeared to influence knowledge of contraceptives and pregnancy-related issues. It was revealed that participants who indicated that they were married were more likely to be knowledgeable about pregnancy and contraceptive methods than those who indicated that they were single. This finding partly substantiates that of another study regarding the high level of SRH knowledge among married compared to single persons [66]. Persons who are single and deaf do not have access to SRH because they engage less with these services, and policies do not generally target their needs. Thus, if SRH services are made readily available to them, their knowledge might improve. This finding suggests that policymakers might need to tailor SRH policies to deaf persons and consider targeted training on contraceptives for single people and young adolescents in the deaf community.

Religiosity tends to influence cultural beliefs in a country, irrespective of religion. Indeed, SRH (specifically contraceptive use and abortion) is highly dictated by religious beliefs and practices associated with cultural norms and values [30, 34, 67, 68]. In this study, religion also emerged as instrumental in understanding the participants’ perceptions of SRH. Differences were found among the participants in terms of religion and knowledge of pregnancy and abortion. These findings are partly consistent with those of other studies on culture and religious beliefs as central to perceptions regarding SRH [34, 37, 39]. In particular, the participants’ understanding regarding abortion was borne out of their religious beliefs, which made it immoral to support even safe abortion. This was expected because religion and culture are intertwined. As people are more religious, their values and normative beliefs influence their understanding of pertinent issues. Although the study did not consider explanations for the differences among the participants in relation to religion, it was evident that any SRH intervention that did not take religion and culture into account was probably bound to fail. Therefore, future policy initiatives on SRH could consider a strong partnership between policymakers and religious bodies on awareness creation and education among deaf persons.

The likelihood of more young deaf persons being engaged in abortion was supported by both quantitative and qualitative data. According to both male and female deaf persons, young deaf women are more likely to engage in abortion than girls because of the sexual edge among the youth. This finding is partly consistent with that of other studies on the greater exposure of young girls to poor SRH practices than older women [39, 66]. Once again, culture and stereotypes in society could help explain this trend. Traditionally, marriage has been cherished [40], and children born out of wedlock have been perceived as illegitimate and, sometimes, do not count as equal members of the family. This understanding was apparent among the young and unmarried deaf women, who would be exploring avenues for abortion [30, 48]. With abortion considered illegal in Ghana [48] and potential communication problems at health facilities [43, 45], deaf women may consider adopting self-care practices to terminate their pregnancies. This could further expose them to health risks and even death. This finding points to the urgency for more education among deaf women on contraceptive methods to enable them to protect themselves from unwanted pregnancies.

## Study limitation

The findings of this study should be interpreted with some caution due to some limitations. First, the participants were drawn from the registered membership list of the GNAD; therefore, it is possible that deaf persons who are not GNAD members could have alternative views and knowledge of contraceptive methods, pregnancy-related issues and abortion-related issues. However, as a recognised body, the GNAD is involved in advocating improvements in the well-being of deaf people. Therefore, it was extremely important that the research team collaborate with them in conducting the study. Most importantly, the participants were recruited from diverse backgrounds, thereby reflecting diversity within the members of the association. Second, the research team did not have the capacity to conduct a hearing assessment to determine whether the participants were indeed deaf or had other comorbidities. However, the research team had one-on-one encounters with the participants and were confident that the right participants were recruited for this study. Also, the study objective and potential benefits were explained to the research participants. This helped ensure that the participants understood the study and provided appropriate responses to the questions. Furthermore, the study relied on the accounts of deaf persons only as it was beyond the scope to include the voices of health professionals. Thus, future studies may consider understanding the experiences of health professionals when it comes to the provision of SRH services to deaf persons. This would help expand the debate and provide useful information which may impact policy reforms in Ghana. Overall, a major strength of this study was the recruitment of male and female deaf persons to share their perspectives on the sensitive topics under study.

## Conclusion and study implications

The purpose of this study was to develop a deep understanding of the knowledge of deaf persons regarding SRH. This was achieved by the sequential collection of quantitative and qualitative data from male and female deaf persons as well as triangulation between the quantitative and qualitative data. Furthermore, the study found potential barriers faced by deaf persons in their effort to access SRH information. This spanned education, poverty, ignorance, and limited avenues to receiving SRH information. Although these barriers are similar to what has been reported in the literature [35, 46–51], being deaf places an additional burden on people’s understanding of SRH. Stereotypes and the inability of health professionals to use sign language affected access to SRH information [43], pointing to the need to further rethink the development of affirmative policies that consider the needs and uniqueness of deaf persons.

There are lapses in the current SRH policy framework [29, 48], which make it difficult for deaf persons to access necessary information. Thus, there is a need for health policymakers to design robust policies tailored to suit the uniqueness of deaf persons. The findings of this study have implications for future policymaking and practice in terms of resource mobilization, advocacy and civil society and community. In the short term, health policymakers could consider the solicitation of ideas or engagement with religious bodies on their concerns regarding future SRH reforms. This engagement could be extended to the leadership of the GNAD to elicit their views, needs and services recommendations for inclusion in an SRH policy designed for all. The role of various partners could be made clear and could be integrated in future SRH policies. Also, policymakers and health service providers could expedite awareness creation among the deaf population on various SRH issues. This could be achieved through media campaigns on SRH and professional development for leaders, sensitization campaigns targeting GNAD members and schools for the deaf. The ability of policymakers to partner with schools and the GNAD would help reach a sizeable number of deaf persons as well as training and awareness programmes tailored to various age groups of deaf persons. Moreover, the GNAD leadership could consider submitting proposals, petitions or position papers to health authorities about the exclusion of deaf people from SRH services. This could enable authorities to be aware of the experiences of deaf persons and consider engaging them on ways to improve SRH access.

In the long term, there is a need to develop SRH policies that consider the needs, characteristics and communication needs of deaf persons. Future SRH policy could make provisions for mandatory training and regular in-service training in sign language for health professionals. Consideration could be given to the addition of a sign language course for pre-service health programmes in Ghana. This would equip health professionals with the necessary skills to communicate with deaf persons. Additionally, the government could consider enrolling deaf persons into the national health insurance scheme and make SRH services freely available to them. In summary, access to SRH is a health right, and thus, it is unacceptable for deaf persons to be denied such access. The measures outlined above could improve or promote equitable access to SRH services for deaf persons. 

## Data Availability

The datasets generated and/or analysed during the current study are not publicly available due to ethical restrictions but are available from the corresponding author on reasonable request.

## Abbreviations

ANOVA: Analysis of variance
GNAD: Ghana National Association of the Deaf
SRH: Sexual and Reproductive Health
